# A PRRSV GP5-Mosaic vaccine: Protection of pigs from challenge and *ex vivo* detection of IFNγ responses against several genotype 2 strains

**DOI:** 10.1371/journal.pone.0208801

**Published:** 2019-01-31

**Authors:** Junru Cui, Caitlin M. O’Connell, Antonio Costa, Yan Pan, Joan A. Smyth, Paulo H. Verardi, Diane J. Burgess, Herbert J. Van Kruiningen, Antonio E. Garmendia

**Affiliations:** 1 Department of Pathobiology and Veterinary Science, College of Agriculture, Health and Natural Resources, University of Connecticut, Storrs, Connecticut, United States of America; 2 Department of Pharmaceutical Sciences, School of Pharmacy, University of Connecticut, Storrs, Connecticut, United States of America; 3 Guangxi Key Laboratory of Animal Vaccines and New Technology, Guangxi Veterinary Research Institute, Nanning, Guangxi, PR China; Laurentian, CANADA

## Abstract

Porcine reproductive and respiratory syndrome virus (PRRSV), is a highly mutable RNA virus that affects swine worldwide and its control is very challenging due to its formidable heterogeneity in the field. In the present study, DNA vaccines constructed with PRRSV GP5-Mosaic sequences were complexed to cationic liposomes and administered to experimental pigs by intradermal and intramuscular injection, followed by three boosters 14, 28 and 42 days later. The GP5-Mosaic vaccine thus formulated was immunogenic and induced protection from challenge in vaccinated pigs comparable to that induced by a wild type (VR2332) GP5 DNA vaccine (GP5-WT). Periodic sampling of blood and testing of vaccine-induced responses followed. Interferon-γ (IFN-γ) mRNA expression by virus-stimulated peripheral blood mononuclear cells (PBMCs) of GP5-Mosaic-vaccinated pigs was significantly higher compared to pigs vaccinated with either GP5-WT or empty vector at 21, 35 and 48 days after vaccination. Cross-reactive cellular responses were also demonstrated in GP5-Mosaic vaccinated pigs after stimulation of PBMCs with divergent strains of PRRSV. Thus, significantly higher levels of IFN-γ mRNA were detected when PBMCs from GP5-Mosaic-vaccinated pigs were stimulated by four Genotype 2 strains (VR2332, NADC9, NADC30 and SDSU73), which have at least 10% difference in GP5 amino acid sequences, while such responses were recorded only upon VR2332 stimulation in GP5-WT-vaccinated pigs. In addition, the levels of virus-specific neutralizing antibodies were higher in GP5-Mosaic or GP5-WT vaccinated pigs than those in vector-control pigs. The experimental pigs vaccinated with either the GP5-Mosaic vaccine or the GP5-WT vaccine were partially protected from challenge with VR2332, as measured by significantly lower viral loads in sera and tissues and lower lung lesion scores than the vector control group. These data demonstrate that the GP5-Mosaic vaccine can induce cross-reactive cellular responses to diverse strains, neutralizing antibodies, and protection in pigs.

## Introduction

Porcine reproductive and respiratory syndrome (PRRS) is the most important infectious disease of swine worldwide, and is characterized by reproductive failure and respiratory disease [1,2]. The disease causes a major negative economic impact in all major pork producing countries. For instance, the annual losses are more than 660 million dollars in the United States alone [3]. The causative pathogen, PRRS virus (PRRSV), is a positive-sense, single-stranded enveloped RNA virus and is classified as a member of the genus *Arterivirus*, family *Arteriviridae*, order *Nidovirales* [4,5]. The viral genome is approximately 15 kb, and encodes 8 structural proteins and 14 non-structural proteins [6–9]. The main challenge with PRRSV is its great genetic and antigenic heterogeneity in the field. There are two main genotypes, Genotype 1 (European type) and Genotype 2 (North American type) [10], with up to 40% difference documented in the whole genome between genotypes, and up to 20% divergence within the same genotype [11]. In addition, the virus is highly immunomodulatory [12,13] which adds another layer of complexity in the efforts to control PRRS.

The control of PRRS in the field relies largely on vaccination with modified-live or killed-virus vaccines [14]. Additionally, autogenous vaccines are used [15]. Unfortunately, currently available vaccines only confer protection against homologous strains and possess weak or no ability to provide cross-protection to heterologous strains which are circulating in the field and constantly mutating. This high genetic and antigenic diversity is a major obstacle for the control of PRRS. Vaccines that can provide cross-protection are urgently needed and significant research efforts are being directed to verify the spectrum of protection that can be afforded by either commercial [16] or experimental vaccines. For the latter, several different approaches are being investigated, including consensus sequence vaccines [17–19], multi-subunit vaccines [20–23], molecular breeding by DNA shuffling, recombinant adjuvanted vaccines [24–28] and mosaic T-cell epitope vaccines [29–35]. These approaches are aimed at providing expanded vaccine breadth and depth for highly variable viruses. In a previous study, we have shown that a GP5-Mosaic vaccine designed using the Mosaic Vaccine Tool Suite originally developed for HIV at the Los Alamos National Laboratory (Los Alamos, NM) [29], was immunogenic and conferred partial protection in pigs when delivered as a DNA vaccine [35].

To further improve the performance of this vaccine and test the breadth and depth of the immune responses, we sought to enhance the immune response induced by our vaccine. Recent studies have shown that delivery of DNA vaccines in liposomes resulted in improved DNA uptake and higher levels of antigen expression in different animal models, which range from mouse to non-human primates [36–38]. In this study, the GP5-Mosaic vaccine was complexed to cationic liposomes for delivery into pigs. The ability of the vaccine thus formulated to induce broad cross-reactive cellular responses was tested *ex-vivo* using four Genotype 2 strains diverging with one another in amino acid sequence by at least 10%. The results of vaccine-induced responses and protection afforded to vaccinated pigs against disease challenge are described.

## Materials and methods

### Viruses and cells

PRRSV strains NADC9 (AF396838.1), NADC30 (JN654459.1), SDSU73 (JN654458.1) were kindly provided by Drs. Kay Faaberg and Kelly Lager at USDA ARS. Viruses were propagated in MARC-145 cells. The cells were cultured in Dulbecco’s modified Eagle’s medium (DMEM) supplemented with 10% fetal bovine serum (FBS), 2 mM L-glutamine, 100 U penicillin/mL, and 100 μg streptomycin/mL. Virus titers were calculated using the Reed and Muench method [39]. The viruses were purified over continuous cesium chloride gradients, quantified using a spectrophotometer (NanoDrop 1000, Thermo Scientific) and stored at -80°C until use. Purified VR2332 (EF536003.1) was used as antigen for indirect enzyme linked immunosorbent assay (ELISA). Titrated viruses were used for neutralization assay, recall immune response assay, and challenge (VR2332).

### Protein sequence alignment and phylogenetic analysis

The protein sequence alignments between GP5 sequences of VR2332, NADC9, NADC30, SDSU73, Mosaic 1 and Mosaic 2 and phylogenetic tree were evaluated using CLC Sequence Viewer 8.0 (QIAGEN).

### Preparation of liposomes and formulation with vaccine DNA

Liposomes were prepared by the thin-film hydration method [40]. Briefly, 1,2-dioleoyl-sn-glycero-3-phosphoethanolamine (DOPE), 1,2-dipalmitoyl-sn-glycero-3-phospho-(1'-rac-glycerol) (DPPG) and cholesterol (8:1:1 molar ratio) (Avanti Polar Lipids, Inc. Alabaster, AL) were mixed and dissolved in chloroform (Fisher Scientific Pittsburgh, PA). The lipid/chloroform solution was evaporated using a Buchi Rotavapor followed by being placed under vacuum for 12 hours. Phosphate buffer (pH 6.5), made isotonic with RNase-free sucrose (MP Biomedicals), was then added to the dry lipid film. The lipid film was hydrated with the buffer at <10°C with intermittent sonication (10–20 second durations) until all of the lipid was dissolved. The liposomes were then subjected to two freeze-thaw cycles (i.e. liquid nitrogen for 5 minutes followed by 55°C for 10 minutes). Lastly, the liposomes were extruded using a LIPEX extruder (Northern Lipids Inc., Canada) eight times through a 200-nm polycarbonate membrane (Whatman Nuclepore Track-Etched Membranes) and one time through a stacked 150 nm and 50 nm polycarbonate membranes at 250 psi. Chitosan oligosaccharide solution (50 μM, MW 5k) was slowly added to the liposomal dispersion (250 μM) to form a diluted dispersion of liposomes coated with chitosan. The coated liposomes were then slowly mixed with plasmid DNA (pDNA) (54 μg/mL) to form a liposome-pDNA complex. The complexes were then concentrated using a Vivaflow 50R crossflow filter (Sartorius). The final concentrations were 1 mM total lipid, 100 μM chitosan and 0.54 mg/mL pDNA. The particle size and zeta-potential were measured for each stage of the coating process using a Malvern Zetasizer Nano ZS90. The liposomal dispersions were diluted approximately 50 times prior to the measurement. All measurements were conducted at 25°C, in triplicate, and were reported as mean ± StDev (Z-Ave ± distribution width for particle size) and mean ± StDev (zeta-potential ± mean of zeta-deviation).

### Vaccination of pigs and collection of samples

All the animal work was done under a protocol approved by the University of Connecticut’s Institutional Animal Care and Use Committee. Three to four-week-old, PRRSV-free, porcine circovirus-2-free, cross-bred piglets were used in this study. The GP5-Mosaic vaccine [30], GP5-WT or vector control were delivered as liposome/pDNA complexes. Briefly, the liposome/DNA-GP5-Mosaic vaccine complexes containing 500 μg/mL of DNA were injected to pigs (n = 4) intradermally (0.1 mL) at the back of the ear and intramuscularly (0.9 mL) in the neck at day 0. Booster vaccinations were given at days 14, 28 and 42. Identical schedules and doses were applied to administer GP5-WT (n = 3) or empty vector to control animals (n = 3). Blood samples were collected at days 0, 7, 14, 21, 28, 35, and 42.

### Recall cellular response to PRRSV

PBMCs collected at days 21, 28 and 35 seeded in 24-well flat-bottom plates (5x10^5^ cells/well) in duplicate were stimulated with 200 TCID_50_ VR2332/well or medium for 48 h at 37°C in a 5% CO_2_ atmosphere. The cells were harvested, and total RNA was extracted for quantitative real-time PCR analysis. The same recall test was also done with PBMCs collected at days 0 and at challenge day except that the cells were stimulated with 200 TCID_50_ of VR2332, NADC9, NADC30 or SDSU73 /well or mock stimulated for 48 h at 37°C in a 5% CO_2_ atmosphere.

### Indirect enzyme-linked immunosorbent assay

Indirect ELISA (iELISA) was performed as described previously [35]. Briefly, CsCl_2_ purified VR2332 was coated onto 96-well plates at a concentration of 2.5 μg/mL overnight at 4°C, and the plates were blocked with 10% dry milk in PBS-T (PBS containing 0.05% Tween-20) for 2 h at room temperature. The plates were washed twice with PBS-T and serum samples diluted 40-fold in PBS-T buffer containing 5% dry milk were added. After 60 min at room temperature, the plates were washed five times with PBS-T buffer and incubated with mouse HRP anti-pig IgG (The Jackson Laboratory, Bar Harbor, ME) in 1:1000 dilution for 60 min at room temperature. The plates were washed five times in PBS-T, substrate was added and incubated in the dark at room temperature for 15 min. The reactions were stopped with 1 M HCl solution. The plates were read at OD_450_ in a microplate reader (Biotek HTK model, Winooski, VT). Archival PRRSV positive and negative pig sera were used as controls in each run. Three repeats were done for statistical analysis. Results shown as fold change in OD absorbance.

### Serum neutralization test

Serum neutralization tests were performed using as reported [35]. Briefly, test sera were mixed with equal volumes of DMEM containing 100 TCID_50_ of VR2332. After incubation at 37°C for 1 h, the serum-virus mixtures were added onto MARC-145 monolayers in 96-well plates and incubated at 37°C in a 5% CO_2_ atmosphere for 48 h (final serum dilution 1:4). VR2332 virus plus negative serum, and uninfected cells, served as virus and cell controls, respectively. The neutralizing capacity of serum from experimental pigs was quantified by measuring viral copy numbers by qRT-PCR in supernatants 48 h after infection of cells with pre-incubated serum-virus mixtures.

### Quantitative real-time PCR

Total RNA was extracted from 250 μL of serum or supernatants of infected cell culture using TRIzol LS Reagent or from tissues using TRIzol Reagent (Invitrogen, Grand Island, NY) according to manufacturer’s instructions. RNA was quantified using a spectrophotometer (NanoDrop 1000, Thermo Scientific). cDNA was synthesized using random primers (Invitrogen, Grand Island, NY) in a 20 μL reaction mixture. The reaction was run in a thermocycler (Applied Biosystems GeneAmp PCR System 2400) as follows: 26°C for 10 min, 42°C for 45 min and 75°C for 10 min. SYBR Green real-time PCR was then performed, SYBR qPCR Master Mix was purchased from Bimake (Bimake, Houston, TX) and the cDNA used as a template and ORF7 F: 5′-AAA TGG GGC TTC TCC GGG TTT T-3′ and ORF7 R: 5′-GCA CAG TAT GAT GCG TAG GC-3′ as the forward and reverse primers for ORF7, respectively. The PCR reaction was performed at 95°C for 2 min, followed by 40 cycles of 95°C for 15 s and 61°C for 1 min using Bio-Rad CFX96 Touch System (Bio-Rad, Hercules, CA). For each assay, a standard curve was generated using serially diluted purified viral RNA which contained 10^2^–10^7^ copies/μL. In each run, positive and negative reference samples were run along with the test samples. The viral loads were determined by plotting the Ct values against the standard curve. Melting curves were analyzed to verify the specificity of the PCR.

To test for cytokine expression, total RNA was extracted from virus-stimulated or mock-treated PBMCs using TRIzol Reagent. cDNA synthesis and real-time PCR followed the same protocol described above. GAPDH (forward primer: 5’-CGT CCC TGA GAC ACG ATG GT- 3’ and reverse primer: 5’-CCC GAT GCG GCC AAA T -3’) was used as internal control to calculate the changes of IFN-γ (forward primer: 5’-TGG TAG CTC TGG GAA ACT GAA TG—3’ and reverse primer: 5’- GGC TTT GCG CTG GAT CTG -3’) or interleukin-10 (IL-10) (forward primer: 5’- TGA GAA CAG CTG CAT CCA CTT C- 3’ and reverse primer: 5’-TCT GGT CCT TCG TTT GAA AGA AA -3’) by the delta-delta method [41].

### Challenge, necropsy, tissue samples, and lung lesion scoring

Virus challenge was at day 48, A total of 10^6^ TCID_50_/pig of VR2332 was given via both intranasal and intramuscular routes, Blood samples were collected the day of challenge, and 4, 7, 11 and 14 days post-challenge. and Necropsies and post mortem examinations were performed at day 62. The lungs were evaluated macroscopically, weighed, and bronchoalveolar lavages (BAL) were then performed. Tissue samples were collected from each lung lobe, tracheobronchial lymph nodes (TBLN), spleen, and inguinal lymph nodes (ILN), both for fixation in 10% neutral buffered formalin or as frozen specimens. Fixed tissues were processed to paraffin embedding, sectioned, and stained with hematoxylin and eosin for histologic evaluation. Lung lesion scoring was done by a board-certified veterinary pathologist, blinded to the treatment groups, on nine lung sections per pig using a scoring system as previously described [42].

### Statistical analysis

Student t-test or Two-way ANOVA was used to evaluate the differences between the samples within or between groups. The data were analyzed using GraphPad Prism (version 7.0).

## Results

### Sequence alignment and analysis of GP5

**Fig 1:**
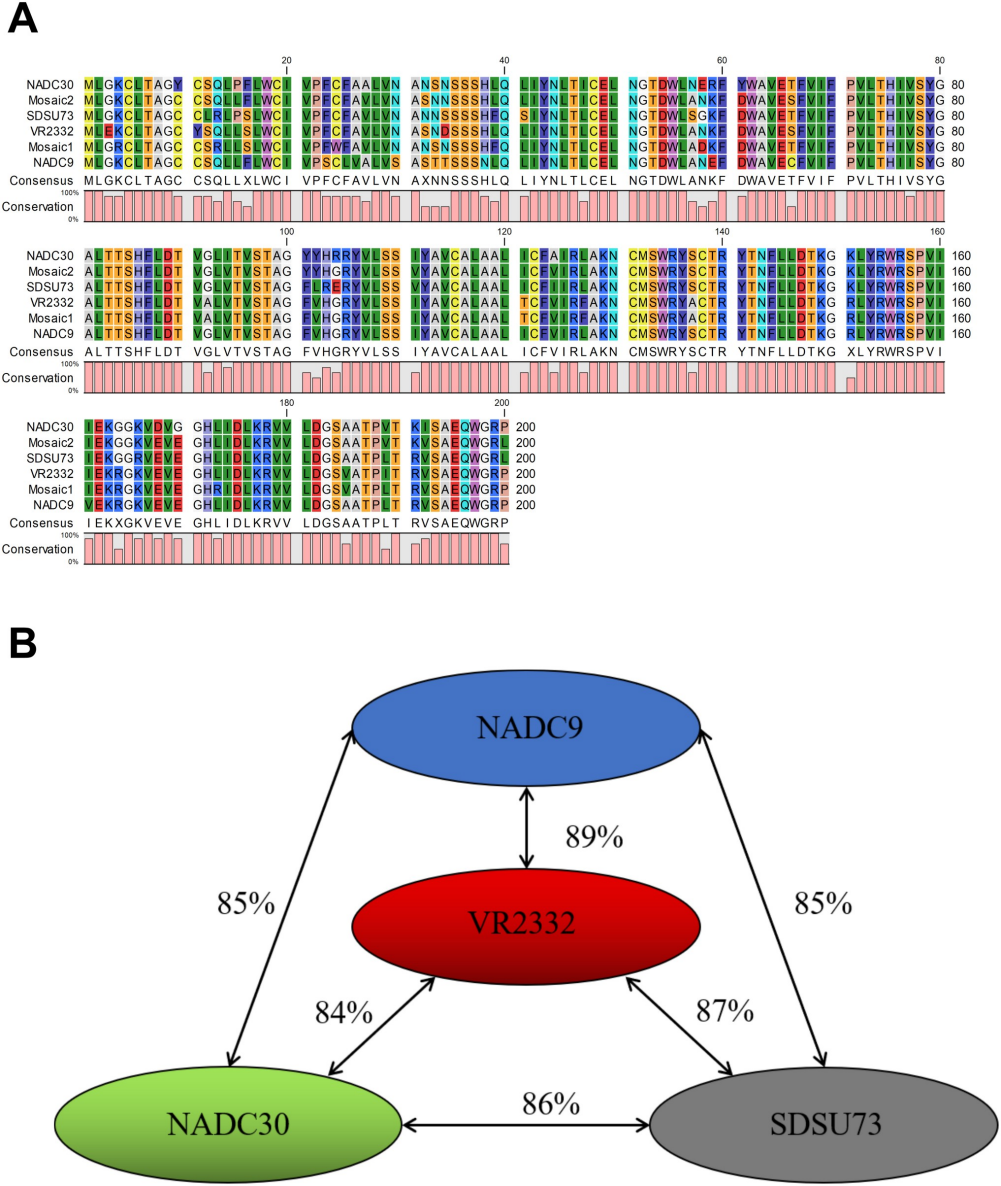
Comparison of amino acid sequences of GP5 of two Mosaic sequences and 4 genotype 2 PRRSV strains. **A.** Alignment of GP5 amino acid sequence; The analysis was done using CLC Sequencer Viewer 8.0. **B.** Percent identities of GP5 amino acid sequences between selected strains used in the study were compared using BLAST.

The GP5 amino acid (aa) sequences of Mosaic 1 and Mosaic 2 of the GP5-Mosaic vaccine [35], NADC9, NADC30 and SDSU73 were of the same size and with no deletions or insertions, when compared to VR2332, the prototype of PRRSV genotype 2 (Fig 1A). Sequence alignments of GP5 showed that the percent aa identities ranged from 84–89% among the four strains used in the study (Fig 1B). Phylogenetic analysis of the above listed strains and mosaics can be found (S1 Fig).

### Size of liposome/DNA complexes

Particle sizes were determined, and the size distribution was very narrow. The size of liposome only was 144.40±1.44 nm, the size of liposome+chitosan was 228.20±4.36 nm, and the size of liposome/pDNA complex was 380.93±6.66 nm (Table 1) a size that is optimum for processing and activation of immune responses.

**Table 1:** Conservation of PRRSV GP5 B-cell/T-cell epitopes.

GP5/PRRSV	B-cell epitope	T-cell epitope 1	T-cell epitope 2
Reference	_37_SHLQLIY	_117_LAALICFVIRLAKNC	_149_KGRLYRWRSPVIVEK
Mosaic 1	_37_SHFQLIY	_117_LAALTCFVIRFAKNC	_149_KGRLYRWRSPVIIEK
Mosaic 2	_37_SHLQLIY	_117_LAALICFVIRLAKNC	_149_KGKLYRWRSPVIIEK
VR2332	_37_SHLQLIY	_117_LAALTCFVIRFAKNC	_149_KGRLYRWRSPVIIEK
NADC9	_37_SNLQLIY	_117_LAALICFVIRLAKNC	_149_KGRLYRWRSPVIVEK
NADC30	_37_SHLQLIY	_117_LAALICFAIRLAKNC	_149_KGKLYRWRSPVIIEK
SDSU73	_37_SHFQSIY	_117_LAALICFIIRLAKNC	_149_KGKLYRWRSPVIIEK
	* - - * -* *	* * * *- **-** - -* * *	* * - * * * * * * ***-* *

*means perfect match, _means substitution

### Vaccination with GP5-Mosaic vaccine activated antibodies and cellular responses

**Fig 2:**
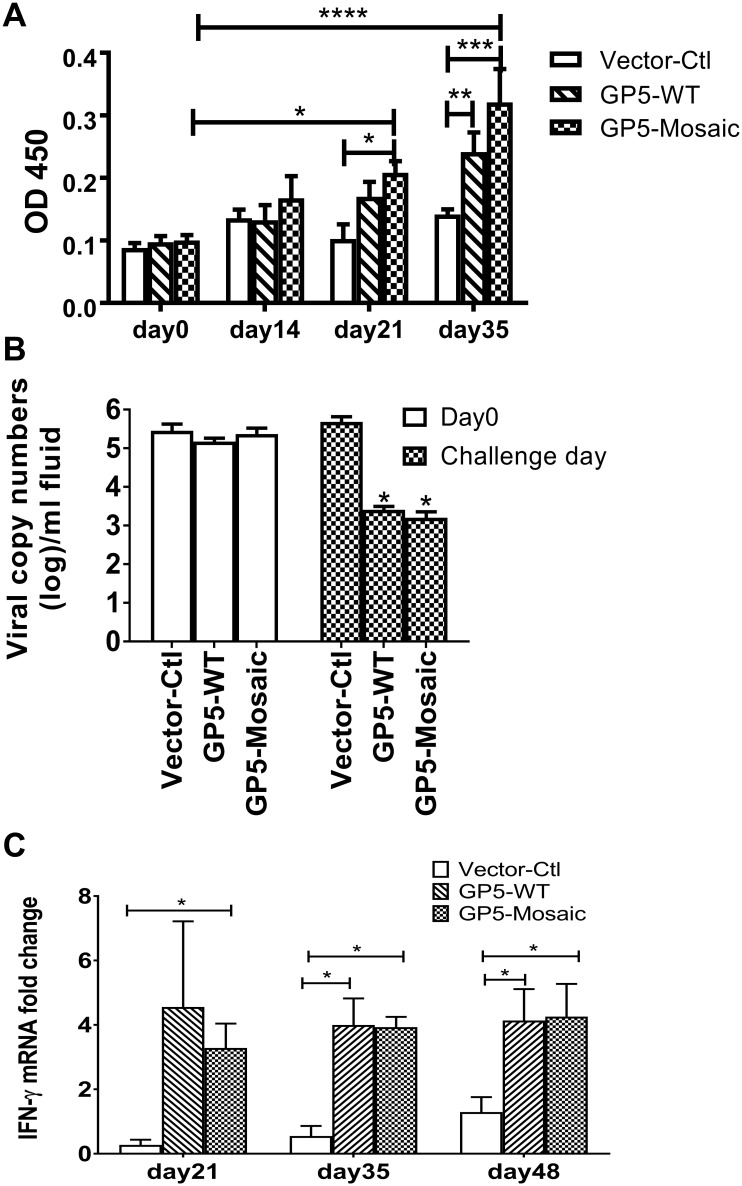
Vaccine-induced humoral and cellular responses. **A.** Virus-specific antibodies detected by iELISA before challenge. **B.** Changes in viral copy numbers (log_10_ scale), to measure of neutralization of virus, as detected by qRT-PCR in cell supernatants after infection with pre-incubated serum-virus mixtures. **C**. IFN-γ mRNA fold changes in VR2332-stimulated PBMCs at days 21, 35 and 48 post immunization. Each bar represents the mean value of each group. Variation is expressed as standard error of the mean. Three experiments were performed independently. Significant differences calculated by a Two-way ANOVA or student t test (*p*<0.05*, *p*<0.01**, *p*<0.001*** or *p*<0.0001****).

The GP5-Mosaic vaccine induced significantly higher levels of antibodies, as measured by iELISA in sera collected at days 21 (*p*<0.05) and 35 (*p*<0.001), (but not at day 14), than those of vector-control animals (Fig 2A). The GP5-WT vaccine induced significantly higher levels of antibodies as detected in sera collected at day 35 compared to those of vector-control animals (*p*<0.01). The sera collected at challenge day from GP5-Mosaic-vaccinated animals neutralized virus, and the levels were significantly higher (*p*<0.01) than those in sera from vector-control animals (Fig 2B). As expected, the ability to induce neutralizing antibodies was also detected in GP5-WT-vaccinated animals. A higher IFN-γ mRNA expression was detected in PBMCs from pigs receiving the GP5-Mosaic vaccine at days 21, 35 and 48 compared to those detected in vector-control pigs (*p*<0.05). Similar differences were also observed between the pigs receiving GP5-WT vaccine and vector-control pigs (Fig 2C).

### Wider cellular responses ex vivo by PBMCs of GP5-Mosaic vaccinated pigs

**Fig 3:**
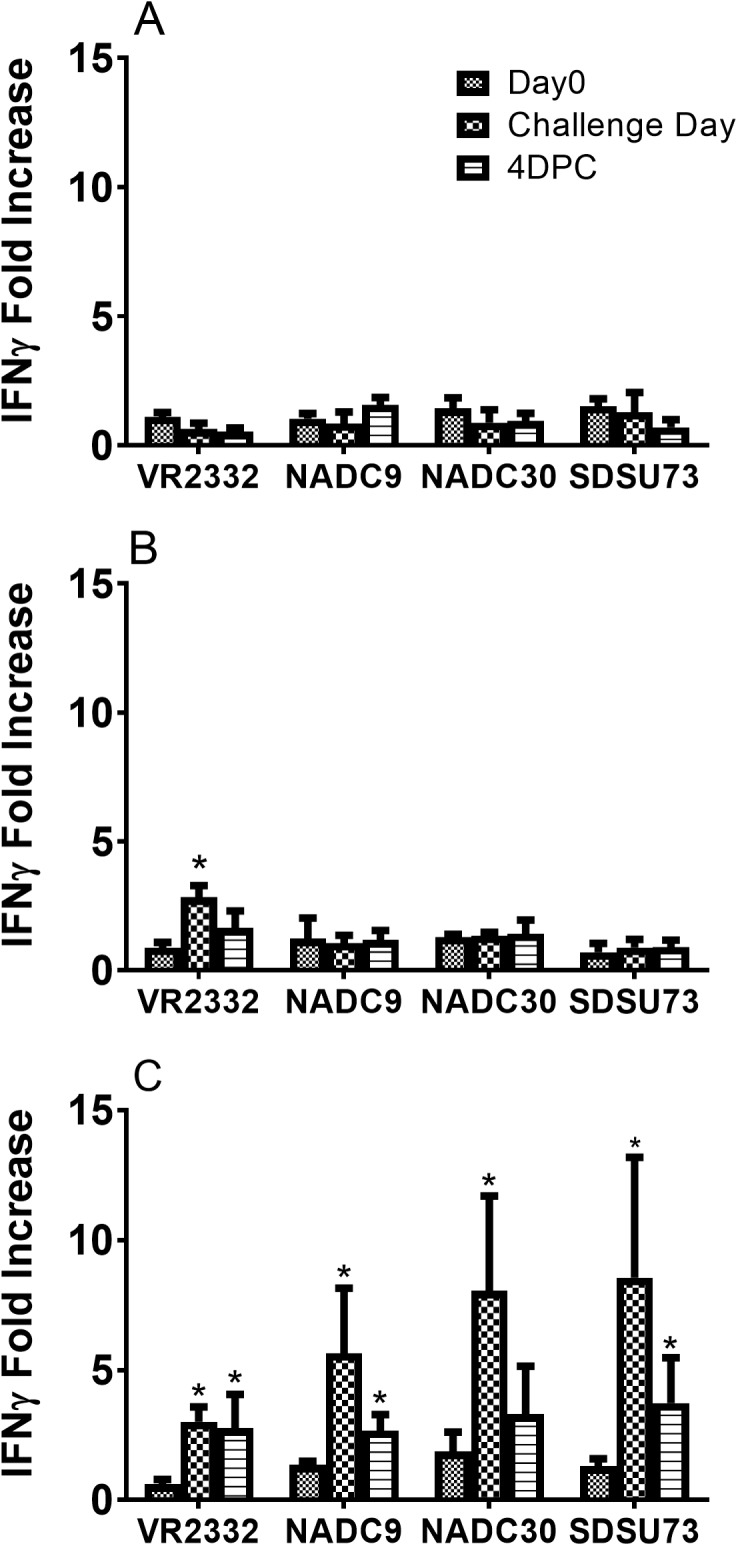
The GP5-Mosaic vaccine induced wider recall cellular responses *ex vivo* than the GP5-WT vaccine. **A.** IFN-γ mRNA fold changes in PBMCs of empty vector control pigs; **B.** IFN-γ mRNA fold changes in PBMCs of GP5-WT vaccinated pigs; **C.** IFN-γ mRNA fold changes in PBMCs of GP5-Mosaic vaccinated pigs. IFN-γ mRNA fold changes in PBMCs at days 0, challenge and 4DPC in response to VR2332, NADC9, NADC30 and SDSU73. Fold increase less than 2 was not considered as real change. Each bar represents the mean value of each group. Variation is expressed as standard error of the mean. Three replicate experiments were performed. Significant differences were calculated by a student t test (*p*<0.05*). (DPC: days post challenge). Significant fold-increases are by comparison with day 0 (asterisks).

*In vitro* stimulation of PBMCs from GP5-Mosaic-vaccinated pigs with four divergent Genotype 2 PRRS virus strains (VR2332, NADC9, NADC30 and SDSU73) revealed broad recall cellular responses with significantly higher levels of IFN-γ mRNA relative fold-change compared to those in mock stimulated controls (*p*<0.05) (Fig 3). In contrast, significantly higher IFN-γ mRNA expression was detected only in VR2332-stimulated PBMCs in GP5-WT-vaccinated pigs compared to mock stimulated controls. No such change was detected in those of vector-control pigs (Fig 3). IL-10 mRNA expression was detected in all groups after challenge with various levels with no defined pattern (S2 Fig).

### Rapid clearance of challenge virus in GP5-Mosaic-vaccinated pigs

**Fig 4:**
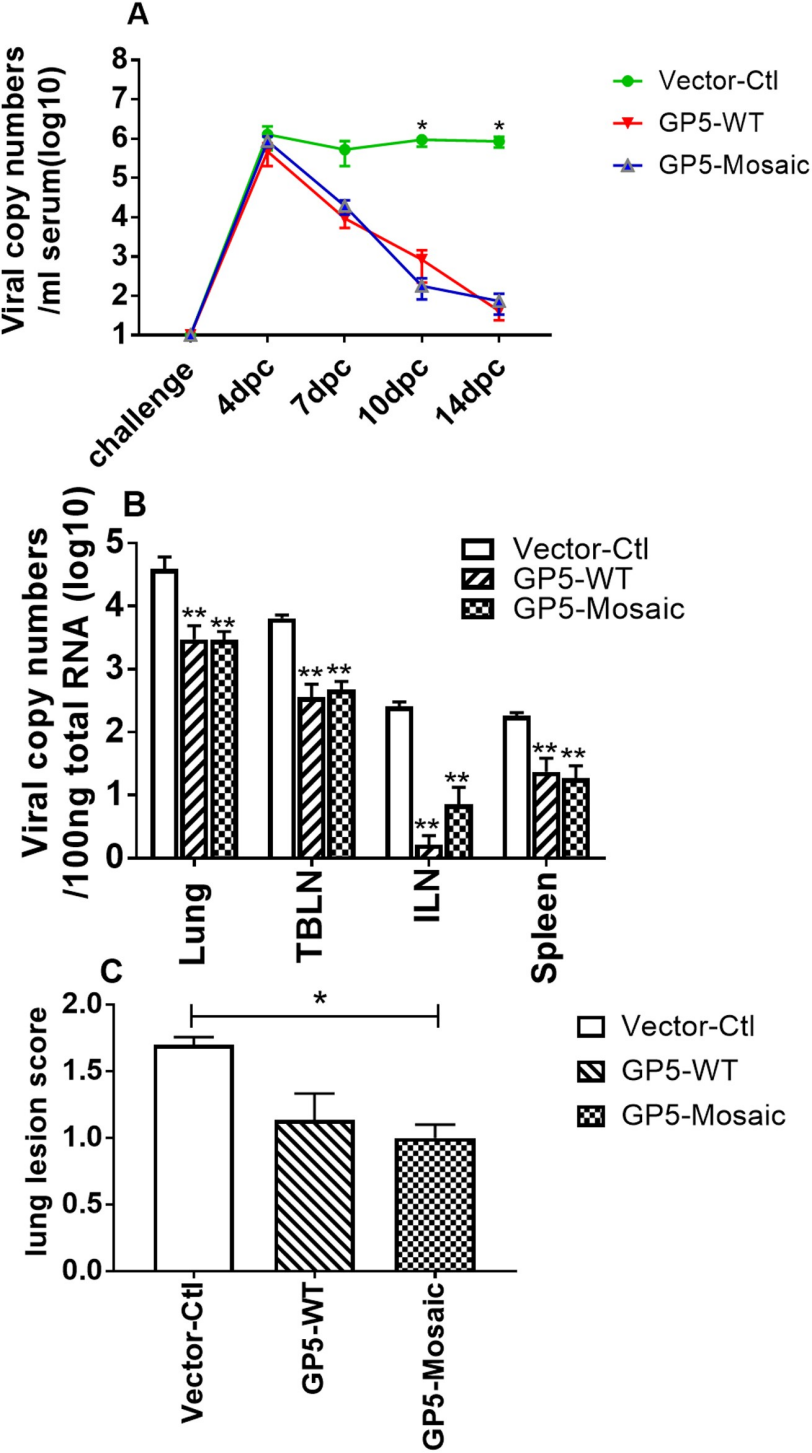
Virus clearance and lung lesion scores. **A.** Viral copy numbers in serum at challenge day and up to 14 days after challenge. **B.** Virus copy numbers in tissues at necropsy (14 DPC) significant differences from vector control (asterisks). **C**. Lung lesion scores. Scores were significantly lower (*p*<0.05) in GP5-Mosaic-vaccinated animals than those in vector-control animals. (DPC: days post challenge).

Viral loads in sera of both GP5-WT and GP5-Mosaic-vaccinated pigs were significantly lower than those in vector-control pigs at 10 and 14 days post challenge (DPC) with VR2332 (*p*<0.05). The GP5-Mosaic vaccine showed a capability in reducing viral loads in serum comparable to the GP5-WT vaccine. Furthermore, viral loads in serum decreased steadily by approximately 4 logs from 4 to 14 DPC in both GP5-WT and GP5-Mosaic-vaccinated pigs while viral loads remained high in the vector-control pigs (Fig 4A). Viral load in tissues including lung, TBLN, spleen and ILN of both GP5-WT and GP5-Mosaic-vaccinated pigs were significantly lower than those in vector-control pigs (*p*<0.01). No significant differences in viral loads between GP5-Mosaic and GP5-WT groups were detected (Fig 4B).

### Lower lung lesion scores detected in GP5-Mosaic-vaccinated animals after challenge

Lung lesion scores were significantly lower in GP5-Mosaic-vaccinated animals after VR2332 challenge than those in vector-control animals when 9 sections of lung were evaluated microscopically (Fig 4C, *p*<0.05). No significant difference was found between GP5-WT and GP5-Mosaic-vaccinated groups.

## Discussion

In order to address the extraordinary diversity of PRRSV, a GP5-Mosaic vaccine was developed in our laboratory and its soundness was confirmed in pigs [35]. The potential of the GP5-Mosaic vaccines to induce cellular responses against heterologous strains was demonstrated in this study. Two GP5-Mosaic sequences and four Genotype 2 PRRSV strains including VR2332, NADC9, NADC30, and SDSU73 which showed at least 10% difference with one another in GP5 aa sequences were used in the study to determine and phylogenetic relationships (S1 Fig). Interestingly, among all the sequences GP5-Mosaic 1 was the closest to VR2332 while GP5-Mosaic 2 was the closest to NADC30, which indicated that the two GP5-Mosaic sequences have broad coverage spanning strains belonging to different lineages. This is supported by the fact that VR2332 and NADC30 were used as the two far ends of these divergent strains with only 84% aa identity between them. Given the sequence data, the ability of the GP5-Mosaic vaccine to induce broader cellular responses than the GP5-WT vaccine, as measured by expression of IFNγ after stimulation of PBMC with the above listed strains, was not totally unexpected.

The GP5-Mosaic vaccine was delivered using cationic liposome/pDNA complexes. Studies have shown that immunization with small liposomes (~100 nm) tended to induce a Th2 response, whereas large liposomes (≥ 400 nm) tended to induce a Th1 response and higher IFN-γ levels [43]. The size of our liposome/pDNA (Liposome/GP5-Mosaic, GP5-WT or vector-control) particles was characterized to be 380.93±6.66 nm (Table 1) which is close to 400 nm, and thus our particles could be considered large liposomes. Indeed the responses induced by the liposome/pDNA complexes were consistent with Th1 responses with higher levels of IFN-γ, which reportedly are significant in protection against PRRSV [44]. Thus, higher levels of IFN-γ mRNA expression were recorded at days 21, 35, and 48 in vaccinated pigs. Vaccination of pigs with the GP5-Mosaic vaccine also induced humoral responses as shown by a steady increase in antibodies over time, and higher levels of neutralizing antibodies before challenge. Interestingly, the GP5-Mosaic vaccine induced even higher levels of antibodies than GP5-WT at day 35, and no significant difference at day 21 compared to vector-control pigs. The use of GP5-Mosaic vaccine as a cocktail of Mosaic 1 and Mosaic 2 may explain, in part, the improvement in vaccine coverage than GP5-WT and the difference in responses.

Mosaic vaccines for HIV-1 have been shown to broaden epitope recognition and increase responses to high-frequency epitopic variants [32,33]. In the present study, the GP5-Mosaic vaccine induced broader cellular responses than GP-WT vaccine, as shown by the significantly higher levels of IFN-γ mRNA expression in response to four divergent Genotype 2 strains tested in recall stimulation of PBMCs. In contrast, PBMCs from GP5-WT-vaccinated pigs only responded to stimulation with the homologous VR2332. No recall responses were detected in the vector-control pigs.

When comparing the conservation of GP5 our T-cell epitopes with two reference ones [45,46], we found that GP5-Mosaic 1 had one perfectly matched T-cell epitope with VR2332, while Mosaic 2 had a perfect match with NADC9 T-cell epitope 1, and Mosaic 2 had a perfect match with NADC30 and SDSU73 T-cell epitope 2 (Table 2). These findings further supported the fact that the GP5-Mosaic vaccine had a broader T-cell epitope identity with all four Genotype 2 PRRSV strains than GP5-WT, which is identical to VR2332. This could explain the broader recall immune responses recorded *ex vivo* with PBMCs from pigs vaccinated with the GP5-Mosaic vaccine. Compared with IFN-γ mRNA expression at challenge day in GP5-Mosaic-vaccinated pigs, lower levels of IFN-γ mRNA were recorded at 4 DPC. This coincided with higher levels of IL-10 mRNA expression recorded at that time point which may have exerted a temporary suppressive effect (S2 Fig). Nevertheless, the GP5-Mosaic vaccine induced much broader cellular responses than GP5-WT vaccine *ex-vivo*. These data support a potential for the GP5-Mosaic vaccine in providing protection of pigs against heterologous PRRSV strains. Herein, the broader cellular responses induced by the GP5-Mosaic vaccine suggest that this vaccine approach expanded both depth and breadth of responses shown as recognition of epitope variants and more epitopes across strains, respectively. Further investigation may identify other potential T-cell epitope(s) that may be included in a mosaic vaccine as data of this study suggest their existence.

**Table 2:** Liposome particle size results.

	Size (d.nm^a^)	StDev	PDI^b^	StDev	Zeta Potential (mV)	StDev
Liposomes Only	144.40	1.44	0.15	0.02	-44.53	19.00
Liposomes+Chitosan	228.20	4.36	0.10	0.03	16.67	4.69
Liposomes + Chitosan + pDNA	380.93	6.66	0.16	0.01	11.80	3.66

^a^d.nm: diameter in nanometers;

^b^PDI refers to polydispersity index.

The rapid virus clearance upon vaccination with either GP5-Mosaic or GP5-WT provides supporting evidence that complexing pDNA to liposomes is an effective way to deliver DNA vaccines. The lower lung lesion scores in GP5-Mosaic-vaccinated animals is further evidence that a measurable level of protection was achieved. Testing protection against additional divergent PRRSV strains will be essential to determine the actual value of the GP5-Mosaic vaccine.

## Supporting information

S1 Fig Phylogenetic analysis based on amino acid sequences of GP5 of two mosaic sequences and 4 genotype II PRRSV strains. The analysis was done using the neighbor-joining method of CLC Sequence Viewer 8.0.
(TIF)

S2 Fig IL-10 fold changes during immunization and after challenge. **A.** IL-10 mRNA fold changes in PBMCs of empty vector control pigs; **B.** IL-10 mRNA fold changes in PBMCs of GP5-WT vaccinated pigs; **C.** IL-10 mRNA fold changes in PBMCs of GP5-Mosaic vaccinated pigs. IL-10 mRNA fold changes in PBMCs at days 0, challenge and 4DPC in response to VR2332, NADC9, NADC30 and SDSU73. Fold increase less than 2 was not considered as real change. Each bar represents the mean value of each group. Variation is expressed as standard error of the mean. Three replicate experiments were performed. Significant differences were calculated by a student t test (*p*<0.05*). (DPC: days post challenge). Significant fold-increases are by comparison with day 0 (asterisks).
(TIF)

S1 Checklist NC3Rs ARRIVE guidelines checklist SUBMITTED. (PDF)
