# Correction: A PRRSV GP5-Mosaic vaccine: Protection of pigs from challenge and *ex vivo* detection of IFNγ responses against several genotype 2 strains

**DOI:** 10.1371/journal.pone.0213091

**Published:** 2019-02-22

**Authors:** Junru Cui, Caitlin M. O’Connell, Antonio Costa, Yan Pan, Joan A. Smyth, Paulo H. Verardi, Diane J. Burgess, Herbert J. Van Kruiningen, Antonio E. Garmendia

The table numbers for Tables 1 and 2 are incorrectly switched. The table that appears as Table 1 should be Table 2, and the table that appears as Table 2 should be Table 1.

**Table 1:** Liposome particle size results.

	Size (d.nm^a^)	StDev	PDI^b^	StDev	Zeta Potential (mV)	StDev
Liposomes Only	144.40	1.44	0.15	0.02	-44.53	19.00
Liposomes+Chitosan	228.20	4.36	0.10	0.03	16.67	4.69
Liposomes + Chitosan + pDNA	380.93	6.66	0.16	0.01	11.80	3.66

^a^d.nm: diameter in nanometers

^b^PDI refers to polydispersity index.

**Table 2:** Conservation of PRRSV GP5 B-cell/T-cell epitopes.

GP5/PRRSV	B-cell epitope	T-cell epitope 1	T-cell epitope 2
Reference	_*37*_*SHLQLIY*	_117_LAALICFVIRLAKNC	_149_KGRLYRWRSPVIVEK
Mosaic 1	_*37*_*SHFQLIY*	_117_LAALTCFVIRFAKNC	_149_KGRLYRWRSPVIIEK
Mosaic 2	_*37*_*SHLQLIY*	_117_LAALICFVIRLAKNC	_149_KGKLYRWRSPVIIEK
VR2332	_*37*_*SHLQLIY*	_117_LAALTCFVIRFAKNC	_149_KGRLYRWRSPVIIEK
NADC9	_*37*_*SNLQLIY*	_117_LAALICFVIRLAKNC	_149_KGRLYRWRSPVIVEK
NADC30	_*37*_*SHLQLIY*	_117_LAALICFAIRLAKNC	_149_KGKLYRWRSPVIIEK
SDSU73	_*37*_*SHFQSIY*	_117_LAALICFIIRLAKNC	_149_KGKLYRWRSPVIIEK
	* - - * -* *	* * * *- **-** - -* * *	* * - * * * * * * ***-* *

*means perfect match, _means substitution
