# Supplementary figures and images for: A PRRSV GP5-Mosaic vaccine: Protection of pigs from challenge and ex vivo detection of IFNγ responses against several genotype 2 strains
Source: PLoS One. 2019 Jan 31;14(1):e0208801. doi: 10.1371/journal.pone.0208801 (PMC6354972; doi:10.1371/journal.pone.0208801)

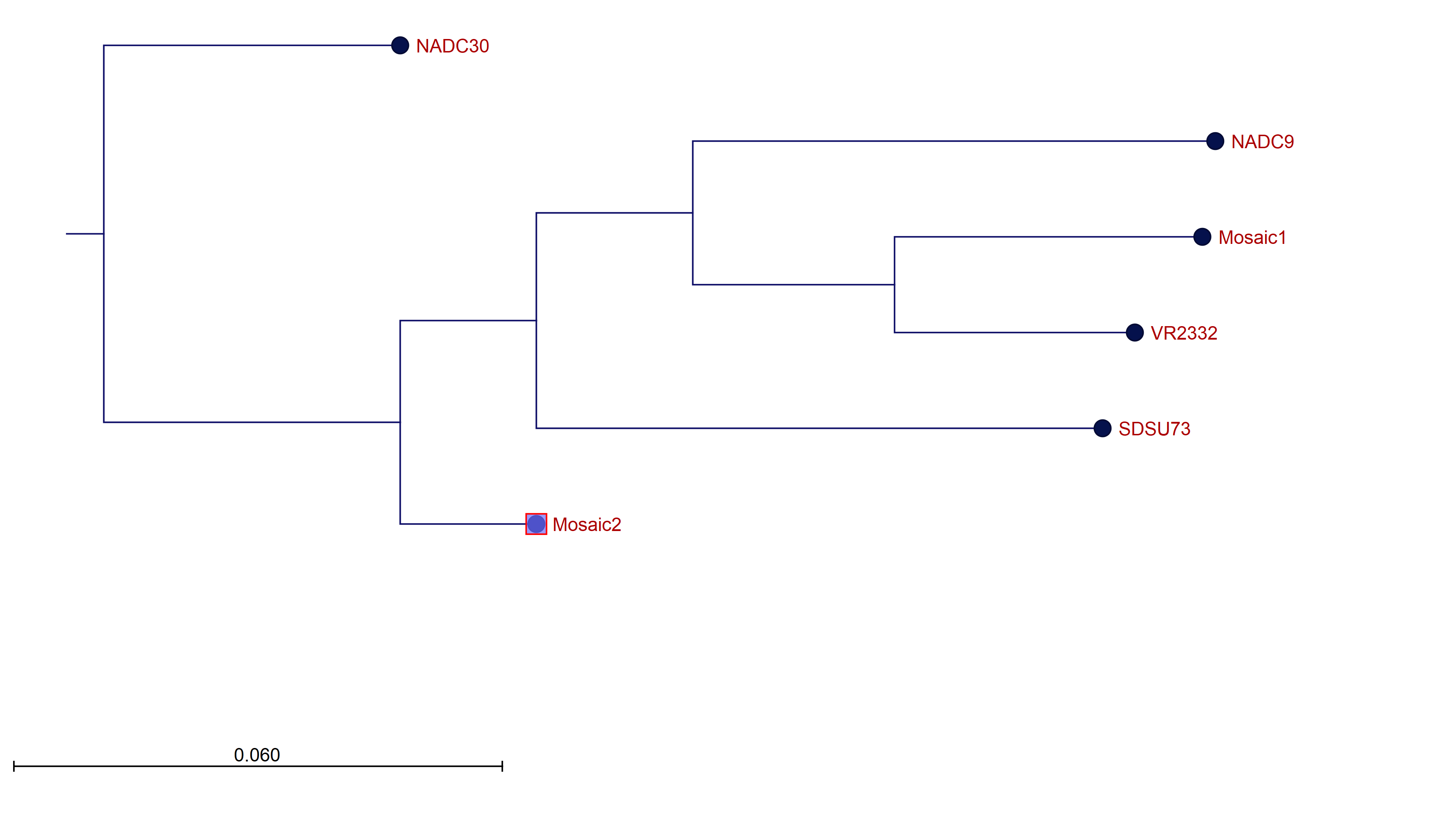

Supplement: S1 Fig — The analysis was done using the neighbor-joining method of CLC Sequence Viewer 8.0. (TIF) [file pone.0208801.s001.tif]

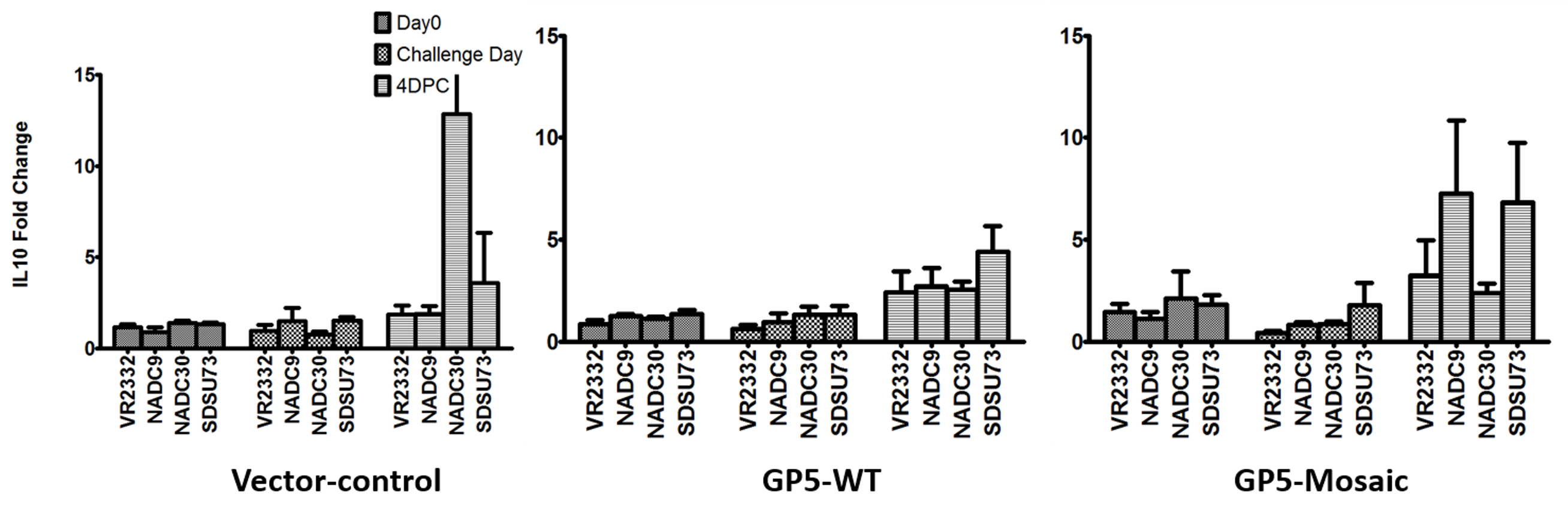

Supplement: S2 Fig — A. IL-10 mRNA fold changes in PBMCs of empty vector control pigs; B. IL-10 mRNA fold changes in PBMCs of GP5-WT vaccinated pigs; C. IL-10 mRNA fold changes in PBMCs of GP5-Mosaic vaccinated pigs. IL-10 mRNA fold changes in PBMCs at days 0, challenge and 4DPC in response to VR2332, NADC9, NADC30 and SDSU73. Fold increase less than 2 was not considered as real change. Each bar represents the mean value of each group. Variation is expressed as standard error of the mean. Three replicate experiments were performed. Significant differences were calculated by a student t test (p<0.05*). (DPC: days post challenge). Significant fold-increases are by comparison with day 0 (asterisks). (TIF) [file pone.0208801.s002.tif]
